# Increased nutrient availability in dense breast tissue of postmenopausal women *in vivo*

**DOI:** 10.1038/srep42733

**Published:** 2017-02-15

**Authors:** Annelie Abrahamsson, Anna Rzepecka, Charlotta Dabrosin

**Affiliations:** 1 Department of Oncology and Department of Clinical and Experimental Medicine, Linköping University, Linköping, Sweden; 2 Department of Radiology and Department of Medical and Health Sciences, Linköping University, Linköping, Sweden

## Abstract

Metabolic reprogramming is a hallmark of cancer. Nutrient availability in the tissue microenvironment determines cellular events and may play a role in breast carcinogenesis. High mammographic density is an independent risk factor for breast cancer. Whether nutrient availability differs in normal breast tissues with various densities is unknown. Therefore we investigated whether breast tissues with various densities exhibited differences in nutrient availability. Healthy postmenopausal women from the regular mammographic screening program who had either predominantly fatty breast tissue (nondense), n = 18, or extremely dense breast tissue (dense), n = 20, were included. Microdialysis was performed for the *in vivo* sampling of amino acids (AAs), analyzed by ultra-high performance liquid chromatography with tandem mass spectroscopy, glucose, lactate and vascular endothelial growth factor (VEGF) in breast tissues and, as a control, in abdominal subcutaneous (s.c.) fat. We found that dense breast tissue exhibited significantly increased levels of 20 proteinogenic AAs and that 18 of these AAs correlated significantly with VEGF. No differences were found in the s.c. fat, except for one AA, suggesting tissue-specific alterations in the breast. Glucose and lactate were unaltered. Our findings provide novel insights into the biology of dense breast tissue that may be explored for breast cancer prevention strategies.

Cancers exhibit different cellular metabolism than do normal differentiated cells and alterations in the metabolism are now recognized as a hallmark of cancer^1^,^2^. Amino acids (AAs), which are the building blocks for proteins including growth factors, play a crucial role in the physiological control of both normal tissues and cancer cells, which have an increased demand for proteinogenic AAs due to accelerated growth and proliferation. Indispensable AAs cannot be synthesized by cells and their uptake from the extracellular environment is a prerequisite for protein biosynthesis and cell viability^3^. Cells may sense the availability of extracellular and intracellular AAs, thereby linking the abundance of AAs to protein synthesis^4^. Additionally, extracellular nutrients can regulate cell signaling without affecting the intracellular levels^5^,^6^. For example, it has been shown that extracellular AAs can activate mechanistic target of rapamycin complex 1 (mTORC1), which is a key regulator of cell growth in response to various nutrient signals, without affecting the intracellular AA levels^6^. Thus, local extracellular levels of proteinogenic AAs in a tissue may play a vital role in controlling the local tissue microenvironment.

Breast cancer affects more than 10% of all women in the Western world and its incidence continues to increase^7^. Mammography screening programs and improved treatments have reduced the death rate for breast cancer, but efficient breast cancer prevention strategies could more effectively reduce the mortality and morbidity associated with this disease^7^. One of the major independent risk factors for breast cancer is dense breast tissue on mammography; women with increased breast density have a four-fold increased risk of developing breast cancer compared to women with entirely fatty breast tissue (nondense)^8^.

Dense breast tissue contains higher amounts of stroma and less fat than nondense breast tissue does but conflicting data regarding the amount of epithelial cells, their proliferation rate, and steroid receptor expression have been obtained^9^. We, and others, have shown that dense breast tissue is associated with increased inflammation^10^,^11^. A stiff microenvironment, such as mammographic dense breast tissue, has also been associated with increased angiogenic signaling including increased levels of vascular endothelial growth factor (VEGF)^12^,^13^.

Anti-estrogen therapies have been shown to reduce the risk of breast cancer by 30–50%^14^. However, side-effects and decreased quality of life are associated with these treatments^14^,^15^. A more precise biological characterization of dense breast tissue is critical for the identification of novel preventive measures for breast cancer. It is not known if normal breast tissues with various densities exhibit differences in metabolic profiles *in vivo*.

Previous studies have revealed that the plasma and saliva levels of free proteinogenic AAs may be altered in patients with different cancers including breast cancer^16^,^17^. Whether nutrient availability varies locally in breast tissues of different densities has not yet been reported.

We hypothesized that the nutrient availability is associated with mammographic density and VEGF in postmenopausal women. Accordingly, to assess this hypothesis we examined the 20 standard proteinogenic AAs, glucose, lactate, and VEGF *in vivo* in breast tissue with various densities in postmenopausal women.

We included healthy postmenopausal women from the regular mammography screening program. Women were selected as having either nondense or extremely dense breast tissue (dense). Extracellular *in vivo* AAs, glucose, lactate, and VEGF were sampled using microdialysis of the breasts. Our data revealed that all 20 measured AAs were significantly increased in dense breast tissue compared with nondense breast tissue but no differences were found in levels of either glucose or lactate levels. Additionally, there was also a significant correlation between VEGF and 18 of the AAs, which suggested that increased vascular permeability could affect the extracellular microenvironment. If the results of this explorative study are confirmed in larger cohorts of women novel strategies aimed at reducing nutrient availability may be explored for breast cancer prevention.

## Results

### Subject characteristics

No significant differences were found in age, BMI, years since menopause, and plasma estradiol levels between the two groups of women, characteristics of the women are shown in Table 1. To explore whether there were any differences present in the extracellular levels of the metabolites in women with different breast densities *per se,* microdialysis reference catheters were inserted in the abdominal subcutaneous fat at the same time as the breast investigation. As shown in Table 1, no differences in the extracellular levels of 19 of the 20 AA, VEGF, glucose, or lactate were detected.

### Increased AA levels in dense breast tissue

**Figure 1:**
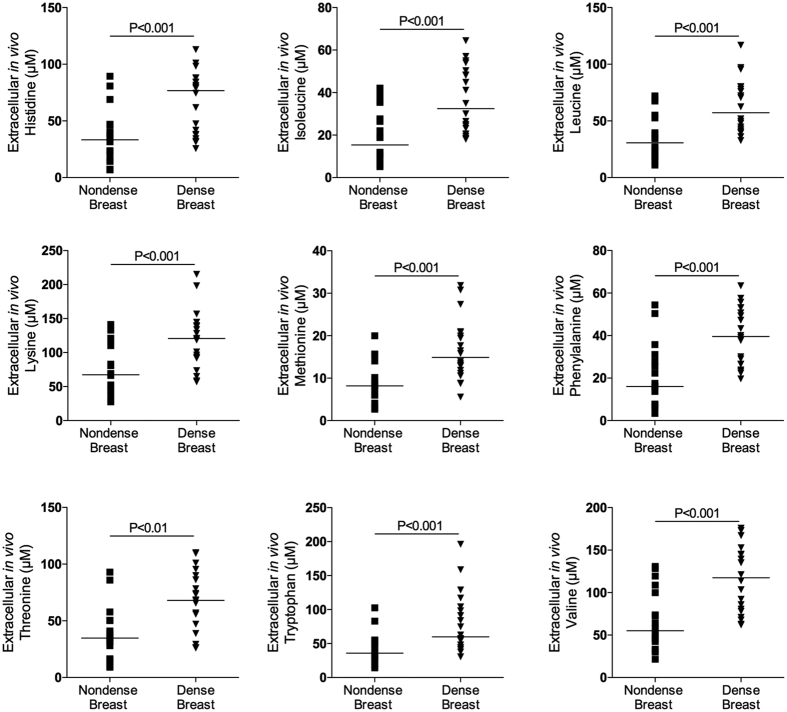
Increased extracellular levels of indispensable amino acids in dense breast tissue *in vivo.* On regular screening mammograms women, who were categorized with breasts characterized as either dense (BI-RADS D) or nondense (BI-RADS A) tissue, were invited to participate in this study as described in the materials and methods. Women with dense (n = 20) and nondense (n = 18) breasts were subjected to microdialysis in the left breast to sample extracellular amino acids. The aligned dot plot shows median line graphics.

**Figure 2:**
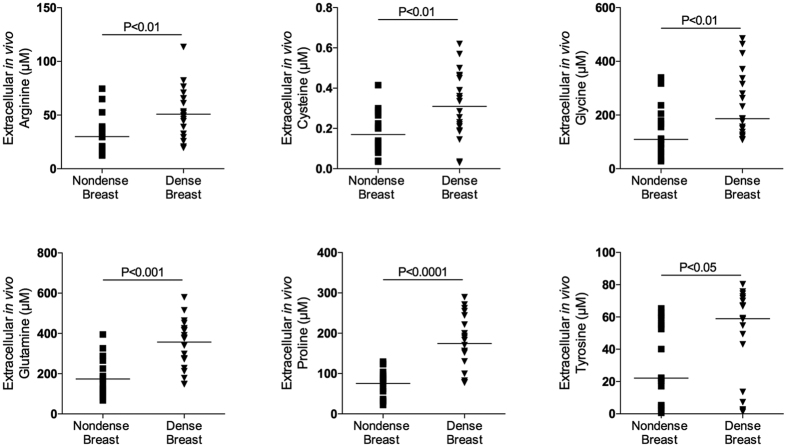
Increased extracellular levels of conditionally indispensable amino acids in dense breast tissue *in vivo.* On regular screening mammograms women, who were categorized with breasts characterized as either dense (BI-RADS D) or nondense (BI-RADS A) tissue, were invited to participate in this study as described in the materials and methods. Women with dense (n = 20) and nondense (n = 18) breasts were subjected to microdialysis in the left breast to sample extracellular amino acids. The aligned dot plot shows median line graphics.

**Figure 3:**
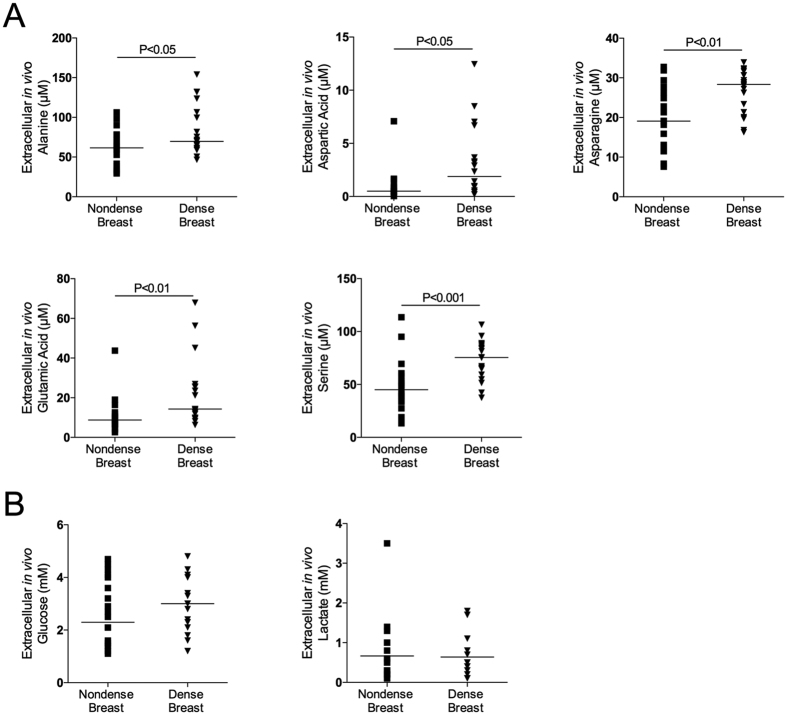
Increased extracellular levels of dispensable amino acids, but not glucose and lactate, in dense breast tissue *in vivo.* On regular screening mammograms women, who were categorized with breasts characterized as either dense (BI-RADS D) or nondense (BI-RADS A) tissue, were invited to participate in this study as described in the materials and methods. Women with dense (n = 20) and nondense (n = 18) breasts were subjected to microdialysis in the left breast to sample extracellular amino acids, glucose and lactate. (**A**) Aligned dot plot with median line graphics of individual amino acids. (**B**) Aligned dot plot with median line graphics of glucose and lactate.

As shown in Figs 1, 2 and 3A there were significantly increased extracellular *in vivo* levels of all types of proteinogenic AAs, i.e., indispensable, conditionally indispensable and dispensable, in dense versus nondense breast tissues. However, the concentrations of the specific AAs varied considerably, ranging from approximately 0.5 μM levels of cysteine to approximately 400 μM of glutamine.

### Glucose and lactate levels in dense and nondense breast tissue

No differences in glucose and lactate levels were detected between dense and nondense breast tissues, Fig. 3B.

### Correlations between VEGF and AA levels

To investigate whether the increases in the AA levels were dependent on increased vascular permeability, the extracellular *in vivo* levels of VEGF were measured. We detected significantly increased level of VEGF in dense breast tissue compared with nondense breast tissue; the concentrations were 8 (2–26) pmol/l in nondense breast compared with 21 (5–50) pmol/l in dense breast tissue, p < 0.001. The data are presented as the median and (range). Indeed, we identified significant correlations between the VEGF levels and any of the AAs except cysteine and tyrosine, as shown in Tables 2, 3 and 4. No correlations were detected between the VEGF levels and either glucose or lactate, as shown in Table 4.

### AAs, glucose, lactate, and VEGF levels in BI-RADS A, fatty breast tissue, and abdominal s.c. fat

As BI-RADS A-classified breasts contain predominantly fat, our study allowed for comparisons of extracellular nutrient availability of fat from different sites in the body. As shown in Table 5 no differences were found between breast tissue from BI-RADS A classified breast and s.c. abdominal fat.

## Discussion

The results of this present study show that dense breast tissue in postmenopausal women was associated with increased AA levels, whereas no differences in glucose and lactate levels were detected between dense and nondense breast tissue. Of the 20 AAs, 18 were significantly correlated with extracellular levels of VEGF, which suggested that increased vascular permeability might affect local levels of AAs in tissues. The increases of AAs were breast tissue specific, except for leucine, as no differences in AA levels were found in abdominal s.c. fat of the two different groups of women.

The abundance of AAs in a tissue may determine the cellular fate. Moreover, it has been shown that an increased supply of AAs from external sources can increase cell proliferation^18^,^19^,^20^. Cells continuously assess the availability of nutrients including AAs to maintain cellular growth and survival. The mTORC1 is a critical signaling hub that regulates both cellular growth and metabolism, and AA uptake is critical for its activation^4^. The AAs leucine and asparagine have been shown to be particularly important for mTORC1 activation^20^,^21^,^22^ and AA abundance in general seems to be a prerequisite for mTORC1 activation^4^. Interestingly, the g-protein coupled taste receptors, T1R1/T1R3, may sense extracellular AA availability and activate mTORC1 without affecting the intracellular levels of AAs, which suggests that cells may turn on their protein production solely by anticipation of an intracellular elevation of AA levels^6^. In addition to cellular uptake of AAs via dedicated transporters, cells have been reported to engulf extracellular fluids in giant vesicles, which contains nutrients that include AAs, via macropinocytosis^23^. Extracellular AAs may enter cells by various mechanisms and thereby affect cellular signaling pathways, which suggests that extracellular AAs may serve as determinants in carcinogenesis. In addition to an ability to turn on pathways such as mTORC1, the increased AA availability may fuel the expansion of any atypical cell and thereby increase the risk of cancer in a tissue. Thus, if atypical cells arise in a dense breast tissue, which contain higher levels of nutrients according to our present data, the extracellular microenvironment would be more permissive for the continuous expansion of these cells. Consequently, the risk of developing a clinically important breast cancer may be increased in dense breast tissue compared to nondense breast tissue, in which the availability of nutrients may be more limited.

Increased tissue stiffness has been found to promote tumor progression via the induction of several cellular functions, including adhesion, integrin expression, cell motility and alternative splicing^24^,^25^,^26^. Mammograhic density has been associated with stiffness and it has indeed been shown that mammographically dense breasts exhibit increased stiffness^13^,^27^. Moreover, breast stiffness *per se* represents an independent risk factor for breast cancer^28^. In experimental studies, the stiffness of the extracellular matrix alone was shown to induce a malignant phenotype of normal mammary epithelial cells^29^. It has also been shown that already aggressive breast cancer cells may show increased motility and proliferation rate in a rigid matrix^12^. Additionally, a stiff microenvironment has also been shown to regulate the angiogenic signaling with increased expression of VEGF in several types of cancer, including breast cancer^12^,^30^,^31^,^32^. In agreement with the results obtained in cancerous tissue, we show in the present study that normal breast tissue may also exhibit differences in VEGF because the extracellular *in vivo* levels of VEGF in mammographic dense breast tissue were almost twice as high as those in nondense breast tissue. Several isoforms of VEGF exist, including freely diffusible VEGF in the extracellular space that is available to endothelial cells, and can exert greater angiogenic properties compared to the heparin bound isoforms that are sequestered in the matrix^33^. In the present study, we sampled freely diffusible VEGF by using microdialysis, a means for the direct quantification of VEGF protein released *in situ* in the bioactive compartment for this protein, as described previously^34^,^35^,^36^,^37^,^38^,^39^,^40^,^41^,^42^. This is a strength of this present study, in addition to the quantification of AAs in the same compartment as VEGF, the extracellular space *in vivo*. VEGF was initially named vascular permeability factor due to its potent ability to increase vascular permeability^43^. Leaky vessels can increase the outflow of compounds from the circulation into the tissue. Our data show strong positive correlations between VEGF and AAs in the tissue, and we speculate that one of the mechanisms involved in this association may be increased vessel permeability induced by VEGF leading to increased AA levels in the tissue. This might also lead to an increase in all molecules from the plasma, but we detected no differences in the extracellular glucose levels between the groups, and no correlation was found between the glucose and VEGF levels. Glucose is the primary nutrient for cellular maintenance and tissue homeostasis. Moreover, the uptake of this vital source of energy is prioritized compared to AA import for protein synthesis. For example, there is a high affinity of glucose for its transporters, which results in the proportional uptake of glucose from extracellular sources^44^. However, the affinity for several AAs to their transporters is lower, which results in the less efficient uptake of AAs into the cells^45^,^46^. Thus, despite increased leakiness in the tissue, a constant uptake of glucose will occur, whereas the lower efficiency of the AA transporters will result in higher extracellular AA levels. There was no difference in extracellular lactate levels between dense and nondense breast tissues, suggesting that no differences existed in tissue hypoxia or basal energy metabolism between the groups.

Mammographic density has been associated with increased risk of breast cancer; women with >75% dense breast area have a four-fold increased risk of developing breast cancer compared to women with <5% dense breast tissue^8^,^9^. It has also been shown that absolute dense area is an independent risk factor for breast cancer whereas absolute nondense area is independently and inversely associated with breast cancer risk^47^. Women with dense breasts may be offered additional screening modalities with higher sensitivity for detection of an already developed breast cancer. However, other interventions aimed at prevention of breast cancer would obviously generate increased survival and less morbidity. Such interventions require knowledge regarding the physiology of normal breast tissue. We believe that our present results reveal previously unrecognized physiological events in breast tissues with different densities.

In summary, in this explorative study we show that the availability of extracellular AAs is increased in dense breast tissue in postmenopausal women. Additionally, dense breast tissue exhibited increased levels of VEGF, and most AAs were significantly correlated with VEGF, which suggests that vascular permeability may affect the extracellular microenvironment. Our study enrolled cohorts of women with breasts who were on either extreme of the mammographic density scale. Our data should be confirmed in a larger cohort that includes women with intermediate breast density and both pre- and postmenopausal women. A more comprehensive analysis of all nutrients available in the extracellular compartment would also be desirable. If confirmed, the nutrient supply in the microenvironment may be included in the search for breast cancer prevention measures.

## Materials and Methods

### Subjects

This study was performed in accordance with the Declaration of Helsinki and was approved by the regional ethical review board of Linköping. All women provided informed written consent. A total of 38 postmenopausal healthy women (55–74 years of age) were included from the screening mammography program at Linköping University Hospital. Exclusion criteria included the following: previous breast cancer, current use of hormone replacement therapy (HRT), any clotting or metabolic disorder, or the use of non-steroidal anti-inflammatory drugs (NSAIDs). A single experienced observer (AR) assessed the mammographic densities according to the Breast Imaging Reporting and Data System (BI-RADS) density scale^48^. Women with BI-RADS A, entirely fatty (nondense) breast tissue, and BI-RADS D, extremely dense breast tissue, were identified. As breast tissue from the different categories, BI-RADS A and BI-RADS D, may contain small areas of dense versus nondense tissue respectively, special care was taken to assess the breast density where the microdialysis catheters were planned to be inserted i.e. only women with BI-RADS A or BI-RADS D in the upper lateral quadrant of the left breast were included. Subsequently, women were invited by letter to participate in this study.

### Microdialysis procedure

Prior to the insertion of the microdialysis catheters, 0.5 ml lidocain (10 mg*/*mL) was administered intracutaneously. One microdialysis catheter was placed in the upper lateral quadrant of the left breast and was directed towards the nipple, and another catheter was inserted in the abdominal subcutaneous fat, as previously described^39^,^49^,^50^,^51^,^52^,^53^. Microdialysis catheters (71*/*M Dialysis AB, Stockholm, Sweden), which consists of a tubular dialysis membrane (length 20 mm, diameter 0.52 mm; atomic mass cut-off 100,000) glued to the end of a double-lumen tube (80 mm long × 0.8 mm in diameter), were inserted via a splitable introducer (M Dialysis AB), connected to a microinfusion pump (M Dialysis AB) and perfused with NaCl 154 mmol*/*L and hydroxyethyl starch 60 g/L (Voluven^®^, Fresenius Kabi, Uppsala, Sweden), at a perfusion rate of 0.5* *μL/min. After a 60-min equilibration period, the outgoing perfusate was stored at −80 °C.

Microdialysis is an *in vivo* sampling technique reflecting the composition of molecules in extracellular compartment of a tissue. The idea of microdialysis is to mimic a blood vessel in an individual organ or tissue. By using microdialysis, a continuous sampling of extracellular molecules by passive diffusion of substances over a semi-permeable membrane takes place. The perfusion fluid leaving the catheters contains the extracellular compounds that have diffused over the membrane and will thus mirror the composition of molecules in extracellular space *in vivo*. Equal volumes from each catheter, collected at the same time point after the insertions of the catheters, were used in all analyses.

### Quantifications of AAs

AAs were measured by ultra-high performance liquid chromatography with tandem mass spectroscopy and electrospray ionization (UHPLC-ESI-MS/MS) following minor modifications of methods as described elsewhere^54^,^55^,^56^,^57^. In brief, the LC-MS/MS system included a PAL autosampler (CTC Analytics, Switzerland) operating at +6 °C, an Advance UHPLC pump, and an EVOQ Elite triple quadrupole mass spectrometer (Bruker Daltonics, Billerica, MA, USA). The ESI source was operated in a positive mode at +4500 V and the source parameters were as follows: probe gas flow 30, nebulizer gas flow 45, probe temperature +400 °C, cone gas flow 15, cone temperature +250 °C, the CID gas was Ar set at 1.5 mTorr. The air and nitrogen gas were provided by Genius 3045 (Peak Scientific Instruments, Inchinnan, Scotland, UK). Mass spectra were scanned in the MRM mode and the optimal precursor/product ions, the collision energy and retention times for the individual AAs and the deuteraded amino acids were used as the internal standards.

A Purospher Star RP-18 endcapped UHPLC column (50 × 2.1 mm, 2 μm, Merck Millipore, Darmstadt, Germany) was used to separate amino acids and the column oven was set to 30 °C. Chemicals and amino acid standards were purchased from Sigma-Aldrich (St. Louis, MO, USA). The LC-MS grade water and acetonitrile, hypergrade for LC-MS LiChrosolv^®^ were obtained from VWR International, Stockholm, Sweden.

Mobile phase A was 0.5 mM perfluoroheptanoic acid (Sigma-Aldrich) in water, the mobile phase B was acetonitrile (ACN). The mobile phase gradient and flow rate were as follows: 0 min, 0% B, 200 μL/min; 1 min, 15% B, 200 μL/min; 2 min, 15% B, 200 μL/min; 5 min, 25% B, 200 μL/min; 9.5 min, 50% B, 200 μL/min; 9.9 min, 80% B, 200 μL/min; 10 min, 0% B, 300 μL/min; 19.9 min, 0% B, 300 μL/min; 22 min, 0% B, 200 μL/min.

5 μl of each microdialysis sample, all collected at the same time point after the insertion of the microdialysis catheters, were diluted with 20 μl LC-MS grade water (Sigma-Aldrich) and stored at −80 °C. On the day of analysis, the samples were thawed at +4 °C, thereafter 10 μl of the sample or the AA standard prepared in 20% Voluven were mixed with 8 μl mobile phase A containing internal standards glycine-D5; DL-glutamic acid-2,4,4-D3; DL-proline-2,3,3,4,4,5,5-D7; Serine-2,3,3-D3; 4-aminobutyric acid-2,2,3,3,4,4-D6 (Cambridge Isotope Laboratories, Tewksbury, MA, USA). The sample injection volume was 10 μl. Standard curves for each AA was created from the known standards included in the analyses and from these standard curves the concentrations of the unknowns were calculated.

### Quantification of glucose, lactate, and VEGF

Glucose and lactate levels were analyzed using the ISCUS^flex^ (M Dialys AB). VEGF was analyzed using the Human Fluorokine MAP kit (R&D Systems, Minneapolis, MN) and analyzed on a Luminex 200 System (Luminex, Austin, TX).

### Statistical analyses

Statistical analyses were performed using GraphPad Prism software 6.0. As not all data were normally distributed nonparametric test were used throughout. Two-tailed Mann Whitney U-test was used when groups were compared and Wilcoxon’s signed rank test was used for pairwise comparisons. Correlations were calculated using the two-tailed Spearman’s rank correlation test. A p-value <0.05 was considered to denote a statically significant difference.

## Additional Information

**How to cite this article**: Abrahamsson, A. *et al*. Increased nutrient availability in dense breast tissue of postmenopausal women *in vivo. Sci. Rep.* **7**, 42733; doi: 10.1038/srep42733 (2017).

**Publisher's note:** Springer Nature remains neutral with regard to jurisdictional claims in published maps and institutional affiliations.

## Figures and Tables

**Table 1:** Characteristics of the included women.

	Nondense Cohort (n = 18)	Dense Cohort (n = 20)	*P*-value
Age (years)	67 (58–73)	62.5 (55–74)	0.2
BMI (kg/m^2^)	25 (19–30)	24.5 (19–32)	0.2
Years since menopause	13 (6–22)	11 (3–21)	0.8
Plasma-estradiol (pM)	112 (22–184)	128 (4–175)	0.9
Alanine	54 (18–119)	59 (17–104)	0.7
Arginine	22 (11–61)	36 (9–71)	0.2
Asparagine	20 (10–28)	21 (7–41)	0.2
Aspartic acid	0.6 (0.02–7)	0.7 (0–1.5)	0.4
Cysteine	0.13 (0.02–0.6)	0.2 (0.01–0.6)	0.2
Glutamine	164 (82–338)	227 (51–450)	0.1
Glutamic acid	6 (2–39)	8 (1–14)	0.3
Glycine	123 (47–356)	160 (47–279)	0.2
Histidine	31 (11–60)	42 (7–76)	0.2
Isoleucine	16 (7–55)	24 (6–49)	0.2
Leucine	27 (14–57)	47 (14–97)	0.003*
Lysine	48 (25–144)	81 (25–204)	0.05
Methionine	7 (4–42)	11 (3–20)	0.5
Phenylalanine	17 (6–46)	25 (4–54)	0.06
Proline	78 (24–173)	92 (10–154)	0.5
Serine	48 (27–145)	53 (16–96)	0.9
Threonine	23 (12–78)	34 (9–95)	0.3
Tryptophan	42 (21–98)	43 (10–99)	0.8
Tyrosine	27 (9–53)	36 (1–74)	0.7
Valine	46 (30–117)	56 (16–126)	0.6
Glucose	2.1 (1.1–5.1)	2.7 (0.4–5.6)	0.5
Lactate	0.2 (0.1–1.5)	0.2 (0–2.6)	0.4
VEGF (pg/ml)	12 (2–37)	16 (4–41)	0.1

Microdialysis was performed in subcutaneous abdominal fat in postmenopausal women who had either nondense (BI-RADS A) or dense (BI-RADS D) breast tissue on their regular screening mammography. VEGF (vascular endothelial growth factor). Values indicate the median (range). Amino acids are shown in μM. Glucose and lactate are shown in mM.

**Table 2:** Spearman’s Correlation Coefficients for *in vivo* extracellular levels of vascular endothelial growth factor (VEGF) and indispensable amino acids in normal breast tissue, n = 38.

	His	Isl	Leu	Lys	Met	Phe	Thr	Trp	Val
VEGF	0.53***	0.57***	0.54***	0.51**	0.60***	0.57***	0.50**	0.41*	0.58***

Significantly different values are indicated in bold.

His, histidine; Isl, isoleucine; Leu, leucine; Lys, lysine; Met, methionine; Phe, phenylalanine; Thr, threonine; Trp, tryptophan; Val, valine. *P < 0.05, **P < 0.01, ***P < 0.001.

**Table 3:** Spearman’s Correlation Coefficients for *in vivo* extracellular levels of vascular endothelial growth factor (VEGF) and conditionally indispensable amino acids in normal breast tissue, n = 38.

	Arginine	Cysteine	Glycine	Glutamine	Proline	Tyrosine
VEGF	0.43**	0.28	0.44**	0.50**	0.46**	0.24

Significantly different values are indicated in bold.

**P < 0.01.

**Table 4:** Spearman’s Correlation Coefficients for *in vivo* extracellular levels of vascular endothelial growth factor (VEGF) and dispensable amino acids, glucose and lactate in normal breast tissue, n = 38. Significantly different values are indicated in bold.

	Alanine	Aspartic acid	Asparagine	Glutamic acid	Serine	Glucose	Lactate
VEGF	0.40*	0.39*	0.58***	0.68***	0.50**	0.25	0.26

*P < 0.05, **P < 0.01, ***P < 0.001.

**Table 5:** Microdialysis was performed in the breast and subcutaneous (s.c.) abdominal fat in postmenopausal women with nondense (BI-RADS A) on their regular screening mammography, n = 18.

	Nondense Breast	Nondense s.c. fat	*P-*value
Alanine	61 (29–106)	54 (18–119)	0.6
Arginine	30 (12–74)	22 (11–61)	0.2
Asparagine	19 (8–33)	20 (10–28)	0.7
Aspartic acid	0.5 (0.04–7)	0.6 (0.02–7)	0.9
Cysteine	0.17 (0.03–0.4)	0.13 (0.02–0.6)	0.4
Glutamine	174 (68–395)	164 (82–338)	0.7
Glutamic acid	9 (2–44)	6 (2–39)	0.4
Glycine	109 (28–339)	123 (47–356)	0.9
Histidine	33 (7–89)	31 (11–60)	0.6
Isoleucine	15 (5–42)	16 (7–55)	0.3
Leucine	30 (11–72)	27 (14–57)	0.2
Lysine	67 (28–141)	48 (25–144)	0.2
Methionine	8 (3–20)	7 (4–42)	0.8
Phenylalanine	16 (3–54)	17 (6–46)	0.4
Proline	82 (22–129)	78 (24–173)	0.2
Serine	45 (13–113)	48 (27–145)	0.9
Threonine	34 (9–93)	23 (12–78)	0.2
Tryptophan	36 (14–102)	42 (21–98)	0.5
Tyrosine	22 (1–65)	27 (9–53)	0.2
Valine	55 (22–130)	46 (30–117)	0.2
Glucose	2.3 (1.1–4.7)	2.1 (1.1–5.1)	0.7
Lactate	0.3 (0.1–3.5)	0.2 (0.1–1.5)	0.1
VEGF (pg/ml)	8 (2–56)	12 (2–37)	0.3

VEGF (vascular endothelial growth factor). Values indicate the median (range). Amino acids are shown in μM. Glucose and lactate are shown in mM.
